# Investigation of the Hydrolytic Degradation Kinetics of 3D-Printed PLA Structures under a Thermally Accelerated Regime

**DOI:** 10.3390/ma17051043

**Published:** 2024-02-24

**Authors:** Bartłomiej Klimczuk, Aleksandra Rudnicka, Oliwia Owczarek, Adam K. Puszkarz, Grzegorz Szparaga, Michał Puchalski

**Affiliations:** 1 ECOResearch Student Research Group, Faculty of Material Technologies and Textile Design, Lodz University of Technology, 116 Zeromskiego Str., 90-924 Lodz, Poland; 2 Division of Materials Science, Commodity Science and Textile Metrology, Textile Institute, Faculty of Material Technologies and Textile Design, Lodz University of Technology, 116 Zeromskiego Str., 90-924 Lodz, Poland; adam.puszkarz@p.lodz.pl; 3 Division of Technology of Yarns, Commodity Science and Textile Metrology, Textile Institute, Faculty of Material Technologies and Textile Design, Lodz University of Technology, 116 Zeromskiego Str., 90-924 Lodz, Poland; grzegorz.szparaga@p.lodz.pl

**Keywords:** polylactide, kinetics, thermal degradation, hydrolytic degradation, 3D printing, mass erosion, micro-CT, viscosimetry, WAXD

## Abstract

The application of biobased and biodegradable polymers, such as polylactide (PLA), in fused deposition modeling (FDM) 3D-printing technology creates a new prospect for rapid prototyping and other applications in the context of ecology. The popularity of the FDM method and its significance in material engineering not only creates new prospects for the development of technical sciences on an industrial scale, but also introduces new technologies into households. In this study, the kinetics of the hydrolytic degradation of samples obtained by the FDM method from commercially available PLA filaments under a thermally accelerated regime were analyzed. The investigation was conducted at the microstructural, supramolecular, and molecular levels by using methods such as micro-computed tomography (micro-CT), wide-angle X-ray diffraction (WAXD), viscosimetry, and mass erosion measurements. The obtained results clearly present the rapid structural changes in 3D-printed materials during degradation due to their amorphous initial structure. The complementary studies carried out at different scale levels allowed us to demonstrate the relationship between the observed structural changes in the samples and the hydrolytic decomposition of the polymer chains, which made it possible to scientifically understand the process and expand the knowledge on biodegradation.

## 1. Introduction

In recent years, eco-friendly trends related to waste reduction and the minimization of the exploitation of non-renewable mineral sources have resulted in the partial replacement of durable and petroleum-based polymers with biodegradable polymers synthesized from renewable sources, such as biomass. A promising alternative in this regard is thermoplastic biobased aliphatic polyesters [1,2,3]. In this group of polymers, polylactide (PLA) is the most popular material of interest from an application point of view. This thermoplastic aliphatic polyester can be used to produce films [4,5], fibers [6,7], yarns [8,9], nonwovens [10,11], and foams [12], designed for many different applications, ranging from the medical field [13,14,15] to agricultural use [16,17]. Recently, it has also become one of the main materials utilized in the fused deposition modeling (FDM) 3D-printing technology for rapid prototyping and other applications [18]. 

The progress of FDM technology and its popularization, especially in the context of rapid prototyping, have made it one of the main contemporary applications of PLA. Filaments for 3D printing from PLA can be modified by additives [19,20,21], which allows the usage of FDM technology to produce not only simple prototypes for constructions, but also elements of devices and machines [22]. Moreover, PLA and its copolymers can be applied through FDM technology in the preparation of biomaterials, such as scaffolds for medical use that are biocompatible and bioresorbable [23,24,25,26]. 

Considering the ever-increasing popularity of rapid prototyping, a problem that may arise over time is the utilization of waste. The application of PLA in 3D-printing technology is a valuable and sustainable solution. This polymer is obtained from renewable sources and its waste is compostable [27]. Additionally, there are research results proving the possibility of recycling PLA [28].

Despite the extensive research that has already been conducted, scientists are still interested in the subject of PLA degradation. The main points of interest are the impact of environmental conditions on the process and effects of degradation, as well as the structural transformation of polylactide at the molecular and supramolecular levels. The assessment of the biodegradation of plastics could be achieved in different ways. According to ISO 20200-2023 [29] standards, the material is compostable if the degree of biodegradation after exposition to a water-based soil–compost mixture under 58 °C is more than 70% after 45 days [28]. On the other hand, composting can be carried out under natural conditions, and according to the current state of knowledge, compostable materials undergo biodegradation for much longer and the time for their full disintegration can reach up to several years [30,31,32].

The subject of the biodegradation of PLA and other aliphatic polyesters is also scientifically interesting due to the complexity of the process, which involves such phenomena as the processes of hydrolysis [33], crystallization [34], mineralization, and etching by microbial organisms or enzymes [35]. More recently, the topic of degradation in the broadest sense has also included PLA materials produced by 3D-printing technology, in which the effects of degradation, rather than its mechanisms, are mainly evaluated [36,37]. In this paper, the results of the investigation of the hydrolytic degradation kinetics of 3D-printed PLA samples under a thermally accelerated regime are presented. The experiment was carried out on samples obtained by the FDM method from two commercially available filaments. The process of hydrolytic degradation was based on the standard ISO 21701:2019 [38]. The 3D-printed PLA samples were degraded in a water-based medium with pH 3.5 and pH 10 under a temperature condition that was near the cold crystallization point of, 90 °C. The reason behind degrading at an elevated temperature was to accelerate the experiment and to analyze the rapid PLA crystallization during degradation [39]. The experimental part of this work was based on the commonly conducted analysis of the degradation effects, such as the determination of the mass percent remaining after degradation and the assessment of structural changes through advanced analytical techniques—X-ray microtomography and X-ray diffraction. Moreover, the effect of the degradation on the molecular weight of PLA was monitored by measuring the intrinsic viscosity of its solution. These methods allowed us to inspect the kinetics of the hydrolytic degradation of the 3D-printed PLA materials and the structural changes at various scale levels, from macroscopic to molecular, which helped to deepen the understanding of the PLA degradation process and its mechanism.

## 2. Materials and Methods

### 2.1. Three-Dimensionally Printed Materials

Three-dimensional samples with an approximately disc shape (diameter: 20 mm; thickness: 2 mm; and mass: 0.5 g) were obtained from two commercially available PLA filaments with a diameter of 1.75 mm: (1) PLA 1—a transparent filament by Spectrum Filaments (Pecice, Poland); and (2) PLA 2—an emerald filament by Devil Design (Mikolow, Poland). The tested materials ware fabricated using an FDM 3D printer, Ender-3 V2 (Creality 3D Technology Co., Ltd., Shenzhen, China), under the following conditions: melting temperature: 200 °C; nozzle diameter: 0.5 mm; table temperature: 60 °C; and infill density 100%. 

### 2.2. Thermally Supported Hydrolytic Degradation

The hydrolytic degradation process was carried out in a distillate water-based medium with various pH values (3.5 (a water solution of acetic acid) and 10 (a water solution of sodium carbonate)) based on the standard ISO 21701:2019 [38]. The disc-shaped samples with a similar mass, 0.5 g, were degraded in 50 mL of the hydrolytic medium under a constant temperature of 90 °C at different time intervals: 1, 4, 7, 10, 14, 18, 21, 25, 28, 32, and 35 days. 

### 2.3. Mass Loss Kinetics

To determine the mass loss caused by the applied degradation process, each sample was washed in distilled water and weighted using balance PS.R1 (Radwag, Radom, Poland). The mass percent remaining after the time of degradation (**D_t_**) was calculated according to the following equation:

(1) $$D_{t}=\frac{m_{t}}{m_{0}}100\%$$

where **m_0_** and **m_t_** are the masses of samples before and after degradation, respectively.

### 2.4. High-Resolution X-ray Tomography 

To investigate how the applied degradation process affected the changes in the microstructure, the samples were examined using high-resolution X-ray tomography (SkyScan 1272 made by Bruker, Kontich, Belgium). The tested materials in subsequent stages of biodegradation were scanned under identical conditions (X-ray source parameters: high voltage of 50 kV; anode current of 200 µA; pixel size of 7 µm; and 180° rotation with an r step of 0.2° without filter) and compared to the samples before the degradation process.

### 2.5. Measurement of the Intrinsic Viscosity

To understand the effects that the process of hydrolytic degradation has on the PLA’s molecular structure, the changes in its molecular weight were analyzed. The average molecular weight reflects the average length of a polymer chain and is directly linked to the viscosity of its solution. This relation is described by the Mark–Houwink–Sakurada equation:

(2) $$\left[\eta \right]=KM_{\eta }^{\alpha }$$

where **[η]** is the intrinsic viscosity of a polymer solution, **M_η_** is the average molecular weight, and **K** and **α** are constants. These constants are not known for PLA in subsequent stages of degradation, which makes it impossible to accurately calculate **M_η_**. However, the relation expressed by the equation allows to indirectly assess the changes in the molecular weight by observing the kinetics of the estimated intrinsic viscosity as a function of time.

The intrinsic viscosity [η], a diluted polylactide/dichloromethane at different stages of degradation, was estimated using an Ubbelohde viscosimeter (Type 2a.) at 23 ± 0.5 °C.

### 2.6. Wide-Angle X-ray Diffraction

The impact of degradation on the supramolecular structure of the PLA samples was characterized using a X’Pert PRO diffractometer (PANalytical, Almelo, The Netherland) with a CuKα source (λ = 0.154 nm) under the following measurement conditions, which are identical for all samples: a high voltage of 40 kV and an anode current of 30 mA. The semiconductor counter X’Celector was used as the detector. The diffraction profiles for the samples were recorded over a 2θ range of 5°–45° with a step of 0.015°. The supramolecular ordering parameters, such as the crystallinity degree and the average crystallite size, were estimated using the WAXSFIT software 1.0 [40].

## 3. Results and Discussion

### 3.1. Macrostructural and Microstructural Changes

**Figure 1:**
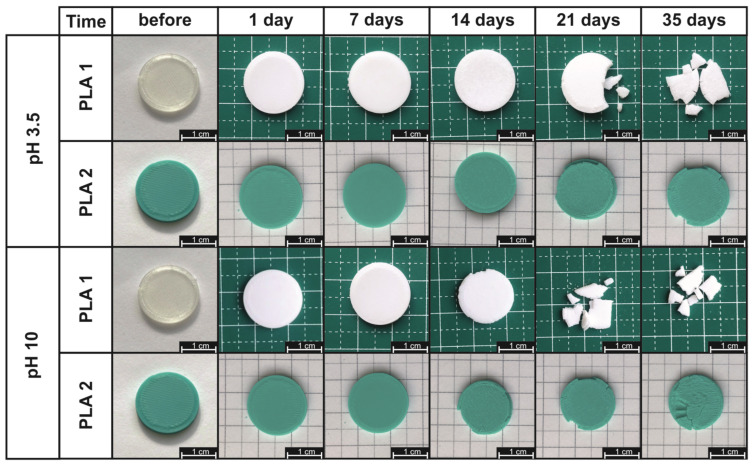
The photographic documentation of the thermally supported hydrolytic degradation of the 3D-printed PLA samples.

The first stage of the characterization of the degradation effects was the analysis of the morphology of the samples after thermally supported degradation. The analysis was carried out on the surface global view by photographic documentation and internal inspection by the micro-CT methods. In Figure 1, the images of the studied samples after the selected degradation steps are presented. According to these photographs, the physical changes in the samples during the thermally supported hydrolytic degradation were similar for both PLA materials, and were mainly observed as the tendency to become brittle that increased with the time of degradation. In addition, it is worth noting that there was a change in the color of the tested materials after just the first day of degradation, which was more significant for the PLA1 sample, which became dull from transparent and glossy. The dulling of the materials after the first day of degradation is probably a result of structural changes, including the crystallization of the polymer, which might have occurred in the case of PLA, especially semicrystalline samples. The further color changes of samples during degradation may additionally be a result of surface erosion. The rapid crystallization of PLA under thermally supported hydrolytic degradation is well known, and for fibrous PLA structures, it was reported in, e.g., Giełdowska et al. (2022) [41].

According to the presented photographs, the samples that were more resistant to the degradation factors were those made of PLA2, for which the effect of fragmentation and tendency to brittleness after 35 days were much lower than those of PLA1. The changes in the samples obtained from PLA2 had mainly surface erosion. The samples printed using the filament PLA1 were characterized by brittleness already after 14–21 days. It is worth noting, however, that the degradation of the tested materials up to day 35 is not complete and can even be considered insignificant. Nonetheless, the presented results show that the degradation rate is clearly influenced by the structure of the tested objects. Porous materials with a high surface-to-volume ratio, such as fibrous products, degrade significantly faster at similar masses [42]. However, 3D prints are solid materials, and the nature of their hydrolytic degradation is a surface erosion phenomenon. 

The photographic documentation presented in Figure 1 also showed a negligible effect of the pH of the applied medium on the rate of degradation evolution of the samples. It can be observed, in the case of the samples made of the PLA1 filament at pH 10, that the samples became brittle already after 14 days, while at pH 3.5, this occurred only after 21 days.

**Figure 2:**
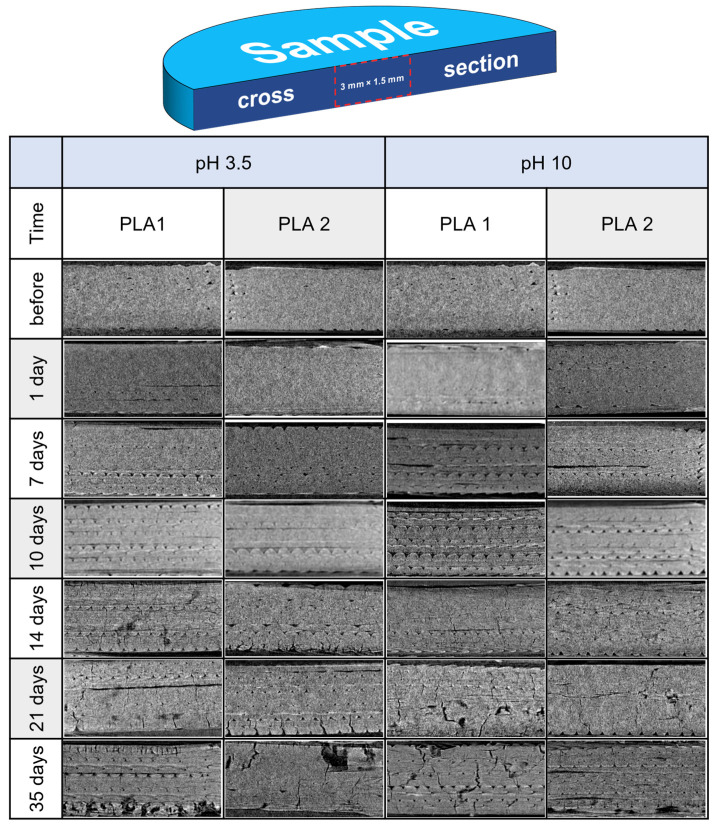
The scheme of the analyzed material fragment selection by means of micro-CT (**top**) and micro-CT cross-section of the tested materials before and after the stages of hydrolytic degradation under the thermally accelerated regime (**bottom**).

The more exhaustive analysis of the morphological structural changes in the 3D-printed PLA samples during degradation were obtained by using computer microtomography. The changes in the structure of the tested materials were analyzed on the basis of a fragment of the cross-section (dimension of 3 mm × 1.5 mm) of the disc-shaped sample, along a plane perpendicular to the disc base passing through the disc axis (scheme shown in Figure 2). The representative results of the microstructure of the samples before and after 1, 7, 14, 21, and 35 days of thermally supported hydrolytic degradation are presented in Figure 2. The PLA 1 and PLA 2 samples before degradation are characterized by a homogeneous microstructure in their cross-section with a few defects due to the raster printing. After the 1st day of the thermally supported hydrological degradation, the first changes are observed as a more visible raster of printing, which is probably the effect of the shrinkage of the PLA. The effect of thermal shrinkage increases, and in the following days, the raster is more visible, which can also be due to erosion not only on the surface of the sample but also in the internal part of the sample in the areas of defects. Additionally, transverse cracks are observed from the 14th day, indicating a tendency to fragmentation of the tested materials. Finally, the disintegration of the samples in both surface and volume is observed after 21 days, and from then onward, the effect keeps increasing.

The micro-CT investigation confirmed the results of the macroscopic observation, where the effect of degradation is more visible for the PLA 1 sample than PLA 2. Moreover, the influence of the pH factor of the degradation medium is insignificant, and slight differences at a favorable pH 10 are seen for the PLA 1 sample.

### 3.2. Mass Loss Kinetics

**Figure 3:**
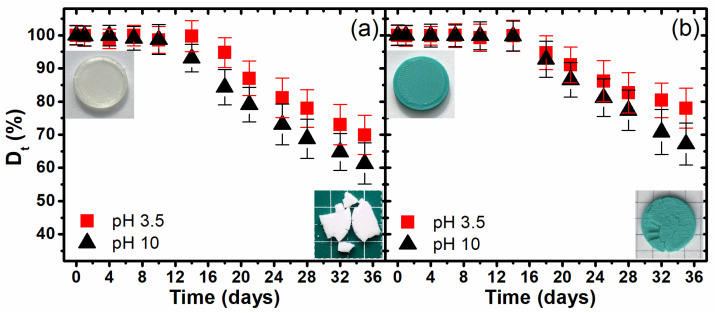
The changes in the mass percent remaining of the investigated 3D-printed samples PLA1 (**a**) and PLA2 (**b**) during hydrolytic degradation under a thermally accelerated regime.

The photographic documentation (Figure 1) and micro-CT images (Figure 2) clearly present the changes in the samples’ morphology, not only at the macroscale and surface view, but also cross-section view during degradation. These results qualitatively indicate the changes in the morphological structure of the materials during the thermally supported hydrological degradation. The next step of the experiment was to examine quantitatively the effects of the degradation. In this regard, a kinetic degradation study was carried out as an analysis of the change in the percentage of remaining mass according to Equation (1) as a function of the degradation time. In Figure 3, the changes in the mass percent remaining (**D_t_**) of the studied 3D-printed samples during the thermally supported hydrolytic degradation in the selected water solution at various pH values are presented. 

A significant mass loss for the samples obtained from both PLA filaments was observed only after the 14th day of degradation, although in the case of the sample obtained from PLA1 degraded in a water-based medium with pH 10, weight loss occurred as early as day 10. These studies indicate that the intensification of the heat factor does not result in rapid degradation and only occurs after the 10th day, which is also confirmed by the photographic documentation and micro-CT images, where the most visible changes were observed after the 14th day of degradation. 

Moreover, the maximum weight loss after 35 days was only 40%—for a sample made of the PLA1 filament and degraded in a water solution with pH 10. Thus, the tested materials are relatively resistant to degradation, which is important when considering them as waste entering into the aquatic environment.

In Table 1, estimated by the least-squares method, two characteristic kinetic parameters that describe the typical erosion profile, the onset time (**t_on_**) and the observed pseudo-first-order rate of erosion constant (**k_e_**), are presented. The **k_e_** is characterized as a slope value according to the following equation [42]:

(3) $$\ln\left(D_{t}\right)=A\;-\;k_{e}t$$

where **D_t_** is the mass percent remaining after the time of degradation estimated according to Equation (1), **t** is the time of degradation starting from the time when the mass loss is significant, and **A** is an intercept. The calculated onset time (**t_on_**) value is derived from intersecting the regression line in Equation (2) with the initial mass value and equal to [42]:

(4) $$t_{\mathrm{on}}=\frac{A\;-\;\ln\left(100\right)}{k_{e}}$$

Considering the small number of data points for each statistical analysis, Table 1 shows a good agreement with Equation (2), with high correlation coefficients (**R**) and relatively small relative standard errors (**SE**) for **k_e_** and intercept values estimated using the OriginPro 2015 software. The analysis of the estimated kinetic parameters of the erosion curve of the 3D-printed PLA samples degraded by thermally supported hydrolysis shows the influence of the pH value of the degradation medium on the pseudo-first-order rate constant and onset time values. The pseudo-first-order rate of the erosion constant (**k_e_**) increased with the increase in the pH value, which was expected. In addition, for a higher medium pH, degradation occurred earlier (with a lower value of **t_on_**), which was definitely seen in the case of PLA1. 

### 3.3. Intrinsic Viscosity Changes

The next type of investigation was the analysis of the kinetics of the degradation of PLA at the molecular level. The intrinsic viscosity of a polymer solution is linked directly to its molecular weight, which reflects the average length of the polymer chains. Therefore, it was possible to assess the changes in the molecular weight of the PLA samples throughout the degradation process by measuring the intrinsic viscosity of their dichloromethane solutions.

**Figure 4:**
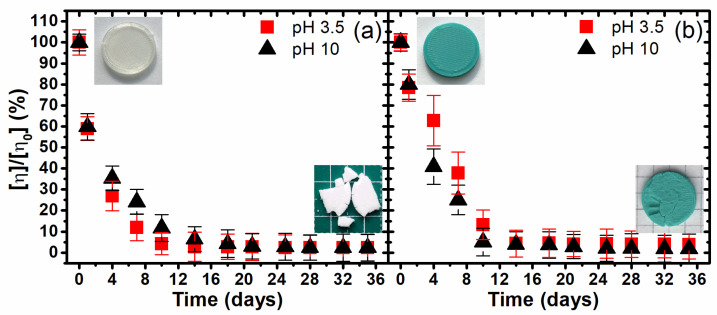
The changes in the intrinsic viscosity of the investigated 3D-printed PLA1 (**a**) and PLA2 (**b**) samples during hydrolytic degradation under a thermally accelerated regime, relative to the initial viscosity of the non-degraded polymer.

Figure 4 shows a very quick intrinsic viscosity decrease at the initial period of both tested samples at both pH values and that decelerates between the 12th and 20th days. Notably, the dynamics of the molecular weight change are definitely greater than their physical weight loss. The noticed phenomenon is extremely important to understand the degradation mechanisms of materials such as 3D-printed PLA structures. The first stage of the changes in the degradation of PLA, as with other plastics, is a reduction in the molecular weight, which in the conducted experiment was the result of the hydrolysis process, which occurred rapidly due to conducting the process at a temperature of 90 °C. A significant mass loss can be observed in samples only after the viscosity, and thus the molecular weight, begin to reach their minimum values. The accelerated mass loss is thus due to the hydrolysis of the shortened PLA chains, resulting in a more frequent release of water-soluble monomers and oligomers. Moreover, at the minimum value of the intrinsic viscosity, i.e., after 14–20 days of degradation, the material became definitely brittle, which in turn confirms that the degradation occurs in amorphous areas and the crystallite edges become structural defects, which is responsible for the poor mechanical properties.

Analogically to the mass erosion analysis, the kinetic parameters of the onset time (**t_on_**) and the pseudo-first-order rate of degradation constant (**k_d_**) were calculated based on the changes in the intrinsic viscosity (Equations (3) and (4), respectively). The mass erosion (Table 1) and the intrinsic viscosity decrease (Table 2) kinetics were calculated and compared to each other as a part of the research. 

Consistent with the data presented in Table 1 and Table 2, the onset time (**t_on_**) for the mass erosion ranges from 10 to almost 14 days and much earlier, 2.1–3.5 days, in the case of the viscosity changes. Any significant mass erosion happens only after nearly all the PLA macromolecules degrade into very short chains or oligomers, which confirmed the proposed mechanism of degradation. Additionally, Table 2 indicates that the PLA samples degrade slightly faster in an alkaline environment. This fact reflects the effects at the macroscopic scale observed on the photographic evidence.

### 3.4. Supramolecular Changes

**Figure 5:**
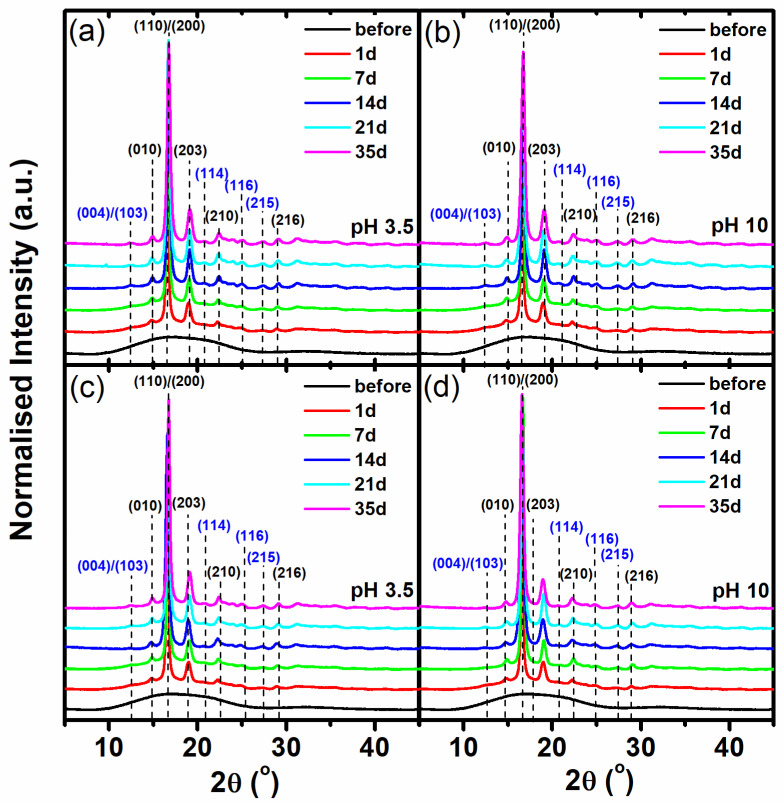
The comparison of the obtained WAXD profiles for the investigated 3D-printed PLA samples, before and after the subsequent stages of hydrolytic degradation under a thermally accelerated regime: (**a**) PLA1—pH 3.5, (**b**) PLA1—pH 10, (**c**) PLA2—pH 3.5, (**d**) PLA2—pH 10.

The last type of investigation was the analysis of the supramolecular structure of PLA by the means of the wide-angle X-ray diffraction method. The comparison of the X-ray diffractograms of the studied 3D-printed PLA samples before and after 1, 7, 14, 21, and 35 days of degradation is shown in Figure 5. The samples before degradation are amorphous, which mainly depends on the methods of PLA processing and whether the samples were obtained without strain, drawing, or thermal conditioning, as crystallization can be induced with combined mechanical and thermal processes [43]. After the first day of thermally supported hydrolytic degradation, all of the studied samples were crystallized, and in all of the obtained diffraction profiles, two dominant diffraction peaks at 2θ 16.5° and 18.8°, corresponding to the (110)/(200) and (203) crystallographic planes, respectively, were observed. In addition, small diffraction peaks were also visible at 14.9°, 22.3°, and 28.8°, which were assigned to reflections from the (010), (210), and (216) lattice planes of the α form of PLA, respectively [33,44,45]. It is worth noting that the effect of the supramolecular changes in the amorphous 3D-printed materials under the accelerated hydrolysis regime is rapid and disregards progressive structural transformation through the formation of the *meso*-phase and α’ form [46], and if these forms appear, they occur before the first 24 h. Observed changes at the supramolecular level explain the apparent microtomographic effects associated with thermal shrinkage. The rapid orientation of the supramolecular structure to the highly crystalline and highly oriented α form causes the sample to undergo thermal shrinkage in the absence of external stress, which is a known consequence of the thermal processing of PLA materials [47,48]. The WAXD results, therefore, are related to the micro-CT observations, in which the shrinkage is visible inside the samples in the first part of the experiment up to the 10th day. This effect is also supported by the extreme decrease in the molecular weight confirmed by the intrinsic viscosity analysis. Short macromolecules are susceptible to crystallization during heat-assisted degradation. In addition, degradation occurring in amorphous areas results in a better presentation of the crystal structure on the X-ray diffraction profiles. Further interesting changes in the supramolecular structure occur after the 10th day of degradation, when diffraction peaks begin to be visible at 2θ 12.4°, 20,6°, 24,9°, and 27.4°, corresponding to the (004)/(103), (114), (116), and (215) crystallographic planes (Figure 4, blue (hkl) marks). The observation of these diffraction peaks is very unique and may indicate the perfectionization of the crystal structure [41]. In our opinion, the perfectionization of the structure observed with the WAXD technique is correlated with the degradation that primarily occurs in amorphous areas. The reduction in the amount of the disordered structure reduces the incoherent scattering of X-rays in these areas, which is visible as an amorphous halo, by causing the diffraction spectra to become the standard of the PLA α form. In the presented experimental results, the standardization of the spectrum can be clearly seen after the beginning of the mass erosion process, which occurred between the 10th and 14th days of degradation (see Figure 3).

The rapid crystallization of the investigated samples during hydrolytic degradation under the thermally accelerated regime was additionally confirmed by the analysis of the crystallinity degree. The crystallinity of the samples was calculated according to Hindeleh and Johnson’s method by means of the WAXSFIT software [49] and the model proposed by Stoclet et al. [45] by the following equation:

(5) $$\chi_{C}=\frac{A_{C}}{A_{C}+A_{A}}\cdot 100\%$$

where **A_A_** and **A_C_** are the integral intensities of the amorphous halo and crystalline peaks, respectively.

**Figure 6:**
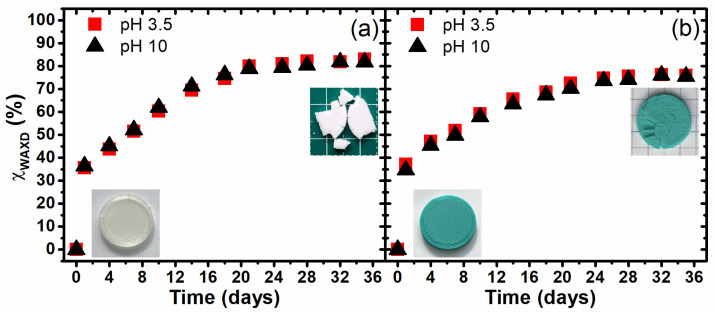
The changes in the crystallinity degree of the investigated 3D-printed PLA1 (**a**) and PLA2 (**b**) samples during hydrolytic degradation under a thermally accelerated regime.

In Figure 6, the increase in the crystallinity degree as the function of the time of degradation is presented. As it is clearly seen, the thermal support of the hydrolytic degradation resulted a rapid crystallization in the first day, when the change in crystallinity from amorphous to the 35–40% crystalline sample was observed. Further changes in the degree of crystallinity were observed, but they were less extreme. The structural transformation occurred in the samples practically until days 20–24, when maximum crystallinity levels were observed for PLA1 (80%) and PLA2 (70%). This part of the experiment revealed how rapid changes occurred in the degradation process and explained the macroscopic effects, such as dulling or internal structure shrinkage. The cracking tendency seen on the computer microtomography image was directly related to the high level of crystallinity. The absence of an amorphous phase completely changes the properties of polymeric materials and they become self-degrading and, in practice, useless. However, it is worth noting that, during the degradation process, the samples lost mass despite their high degree of crystallinity, which showed that even the highly crystalline structure of PLA can be hydrolytically degraded, although it is certainly slower relative to the amorphous structure.

**Figure 7:**
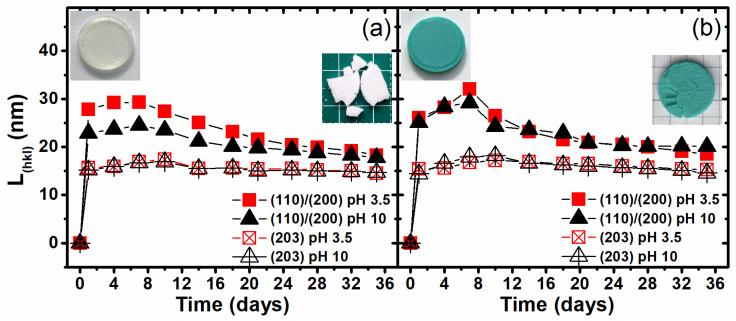
Changes in the crystallite size as a function of the hydrolytic degradation time of the investigated 3D-printed PLA1 (**a**) and PLA2 (**b**) samples during hydrolytic degradation under a thermally accelerated regime.

The perfectionization of the crystal structure brings with it consequences such as the forming of permanent microcrystalites, which are one of the forms of microplastics [41]. The possibilities of creating microcrystalites of PLA during the hydrolytic degradation of the 3D-printed samples were investigated by the numerical analysis of the WAXD diffraction peaks and the determination of the crystalline area size according to Scherrer’s formula:

(6) $$L_{\left(hkl\right)}=\frac{K\lambda }{B\cos\theta }$$

where **L_(hkl)_** is the average crystallite sizes, orthogonal to lattice planes (hkl); **θ** is the Bragg angle for the planes (hkl); **λ** is the wavelength of X-ray radiation (for CuKα λ = 0.154 nm); **B** is the half-width of the diffraction peak for the planes (hkl); and **K** is the Scherrer constant for the particular polymer (0.9 in this case). In Figure 7, the growth in the average crystallites size, orthogonal to the (110)/(200) and (203) lattice planes, is presented. The degradation process under hydrolytic conditions provided the crystallization of PLA with the visible creation of crystallites, and the calculated maximum size observed on the 4th day of degradation was approximately 30 nm for PLA2, degraded in a medium with both pH values, and PLA1, degraded in a medium with pH 3.5, and approximately 25 nm for the PLA1 sample degraded in a medium with pH 10. The crystallite size to the tenths of a micrometer level testifies to the perfection at the unit cell of the PLA supramolecular structure, and the size of the crystalline area suggests the potential for microcrystalite creation in the 3D-printed PLA materials. The creation of crystallites may suggest that permanent and difficult-to-degrade structures are formed, which would suggest the formation of environmentally hazardous microcrystallites. However, as shown in the performed experiment, the average sizes of the crystallites clearly decreased during degradation. Therefore, it can be assumed on the basis of the obtained results that hydrolytic degradation causes the erosion of crystallites, and thus, in the case of PLA, creation and perfection at the ion level of the crystalline phase do not result in the formation of nondegradable structures, which is important from the point of view of environmental impact. Additionally, the degradation of the 3D-printed material in the medium characterized by the various pH values shows the insignificant higher potential for the creation of crystallites during hydrolytic degradation in a medium with pH 3.5 than in that with pH 10. 

## 4. Conclusions

The main goal of this investigation was to present the effect of the hydrolytic degradation of 3D-printed PLA samples under a thermally accelerated regime at the molecular and supramolecular levels with the possibilities of creating microcytsalites. Complementary studies were conducted on various scale levels starting from changes in the macrostructure (macrophotography and mass loss kinetics), changes in the microscale (micro-CT), and effects at the supramolecular level (WAXD) and ending at the changes in the average molecular weight (viscosimetry).

The thermally accelerated hydrolytic degradation experiment based on the ISO standard allowed us to demonstrate the influence of the temperature, or heat transfer, on the kinetics of hydrolytic degradation. In the eroded sample, the decrease in the mass percent remaining and the increase in the crystallinity degree were significantly more rapid than those at lower temperatures, the results of which are described in the cited literature.

From a macroscopic point of view, in all of the studied materials, a change in color was observed after just the first day of degradation, which was significantly more visible for the PLA1 sample, which became dull from transparent and glossy. Additionally, the tendency of materials to become brittle during the time of degradation was also seen. 

A more detailed analysis of the microstructural changes in the samples during the thermally accelerated hydrolytic degradation was carried out by means of micro-computed tomography. The performed analyses clearly showed the internal structural changes in the samples during degradation: firstly, internal shrinkage; secondly, cracking; and finally, erosion were observed. The use of microtomography clearly showed that the degradation process of the PLA materials did not occur only as a surface process, but also the integral degradation of the internal physical structure occurred. The resulting cracks may also suggest the degradation of the amorphous structure not only superficially but also in the interior of the material. This phenomenon is a consequence, in our opinion, of the porosity of structures formed by the 3D-printing method.

On the other hand, the study of the kinetics of the process based on the evolution of the mass percent remaining showed the negligible effect of the pH of the medium on the rate and the time of beginning of the process. In addition, this part of the study also showed slight differences in the degradation rates of the samples made from the two selected filaments. 

The supramolecular structure also changed during the thermally accelerated hydrolytic degradation, and it occurred extremely rapidly. Within a mere 24 h, the amorphous materials became 35–40% crystalline and in a highly ordered α form. The studies using wide-angle X-ray diffraction allowed us to understand the microscopic and macroscopic changes in the samples, which is important to interpret phenomena like shrinkage and cracking. 

The studies of the polymer’s viscosity changes showed a direct correlation between the mass loss and the crystallinity increase during the following days of the experiment and allowed us to better understand the processes taking place during hydrolytic degradation under the thermally accelerated regime. The experiment clearly showed that the first stage of degradation was the decrease in the molecular weight and the crystallization of the shorter macromolecules to the perfect structure of the alpha form. The next stage was the macroscopic changes in the form of material erosion, which was related to the hydrolytic degradation of the amorphous regions.

To summarize the experiment, it should be stated that the 3D-printed samples, despite the applied PLA filaments, were amorphous and, during the degradation process, changed rapidly. The observed changes were accelerated due to the adopted degradation temperature of 90 °C, near to the cold crystallization point. The insignificant influence of the pH of the degradation medium on the degradation kinetics was observed only in the case of observations at the macroscopic scale. The influence of the pH of the medium on the crystallinity degree was marginal, but an insignificant influence on the α form crystal creation was observed. The interesting part of experiment was that we showed the possibility of creating microplastics in the form of crystallites, whose erosion was significantly slower than the degradation of the amorphous form of PLA. The proposed complementary studies clearly showed that the degradation mechanism of the 3D-printed PLA structures under the influence of hydrolysis in the first stage caused significant changes in the molecular structure, which entailed a consequently easier crystallization process, while macroscopic effects were visible only after several days. The presented results also show complementarity in the evaluation of degradation at the molecular and supramolecular levels.

## Figures and Tables

**Table 1:** The kinetic parameters of the evolution of the mass percent remaining of the investigated 3D-printed PLA samples during hydrolytic degradation under a thermally accelerated regime.

Sample	pH of the Medium	A±SE	k_e_ ± SE (days^−1^)	R	t_on_ (days)
PLA1	3.5	4.843 ± 0.018	0.017 ± 0.007	0.989	13.8
PLA1	10	4.801 ± 0.017	0.019 ± 0.006	0.993	10.5
PLA2	3.5	4.864 ± 0.009	0.019 ± 0.004	0.998	13.4
PLA2	10	4.762 ± 0.014	0.012 ± 0.005	0.987	12.9

**Table 2:** The kinetic parameters of the evolution of the intrinsic viscosity decrease experienced by the investigated 3D-printed PLA samples during hydrolytic degradation under a thermally accelerated regime.

Sample	pH of the Medium	A ± SE	k_d_ ± SE (days^−1^)	R	t_on_ (days)
PLA1	3.5	4.843 ± 0.018	0.144 ± 0.013	0.991	2.4
PLA1	10	4.801 ± 0.017	0.145 ± 0.014	0.996	2.1
PLA2	3.5	4.864 ± 0.009	0.146 ± 0.012	0.995	3.0
PLA2	10	4.762 ± 0.014	0.170 ± 0.017	0.985	2.1

## Data Availability

The data presented in this study are available on request from the corresponding author. The data are not publicly available due to restrictions e.g., privacy or ethical..
